# Research on None-Line-of-Sight/Line-of-Sight Identification Method Based on Convolutional Neural Network-Channel Attention Module

**DOI:** 10.3390/s23208552

**Published:** 2023-10-18

**Authors:** Jingjing Zhang, Qingwu Yi, Lu Huang, Zihan Yang, Jianqiang Cheng, Heng Zhang

**Affiliations:** 1 State Key Laboratory of Satellite Navigation System and Equipment Technology, Shijiazhuang 050081, China; zzhangjj1998@163.com (J.Z.); yqw@seu.edu.cn (Q.Y.); yangzh@nuaa.edu.cn (Z.Y.); cjq@seu.edu.cn (J.C.); 230199176@seu.edu.cn (H.Z.); 2 The 54th Research Institute of China Electronics Technology Group Corporation, Shijiazhuang 050081, China; 3 School of Information Science and Engineering, Hebei University of Science and Technology, Shijiazhuang 050018, China

**Keywords:** UWB, NLOS/LOS identification, CNN, CAM, CIR

## Abstract

None-Line-of-Sight (NLOS) propagation of Ultra-Wideband (UWB) signals leads to a decrease in the reliability of positioning accuracy. Therefore, it is essential to identify the channel environment prior to localization to preserve the high-accuracy Line-of-Sight (LOS) ranging results and correct or reject the NLOS ranging results with positive bias. Aiming at the problem of the low accuracy and poor generalization ability of NLOS/LOS identification methods based on Channel Impulse Response (CIR) at present, the multilayer Convolutional Neural Networks (CNN) combined with Channel Attention Module (CAM) for NLOS/LOS identification method is proposed. Firstly, the CAM is embedded in the multilayer CNN to extract the time-domain data features of the original CIR. Then, the global average pooling layer is used to replace the fully connected layer for feature integration and classification output. In addition, the public dataset from the European Horizon 2020 Programme project eWINE is used to perform comparative experiments with different structural models and different identification methods. The results show that the proposed CNN-CAM model has a LOS recall of 92.29%, NLOS recall of 87.71%, accuracy of 90.00%, and F1-score of 90.22%. Compared with the current relatively advanced technology, it has better performance advantages.

## 1. Introduction

With the rapid development of Internet of Things (IoT) technology, intelligent mobile terminal technology, and mobile computing technology, the demand for indoor location services in many industries is getting higher, and the demand for real-time positioning of personnel is becoming more urgent [1,2]. Ultra-wideband (UWB) technology stands out among wireless positioning technologies because of its low power consumption, high-ranging accuracy, high temporal resolution, strong anti-interference capability, etc. [3]. Nevertheless, it is limited by multi-user interference, clock drift, frequency drift, and Non-Line-of-Sight (NLOS) propagation in practical application scenarios [4]. Signal propagation in the NLOS state is affected by obstacles that increase the arrival time, thus causing a positive bias in the distance measurement. It is considered to be one of the main challenges faced by high-precision positioning systems. Therefore, NLOS and Line-of-Sight (LOS) identification before positioning is critical [5].

NLOS/LOS identification methods can be divided into three categories: distance-based estimation methods, position-based estimation methods, and CIR-based estimation methods. The distance-based estimation method performs NLOS and LOS identification by detecting the variance of multiple distance measurements at a given location or by detecting whether the current distance measurement conforms to a specified distribution [6]. The method is relatively simple, but it is limited by the distribution function or time delay. The position-based estimation method performs NLOS/LOS identification during the position-solving process or after the position computation is completed. NLOS can be identified by comparing the position estimates generated using different subsets of the distance estimates when redundant ranging information is present. However, this method is ineffective in the absence of redundant ranging information [7]. If additional environmental information, such as maps, geometric relationships, and path continuity, existed, NLOS identification can be performed using position constraint analysis after the position calculation is completed. However, the disadvantages of external information sources can limit the system’s stability and increase complexity. Over time, CIR-based estimation methods have received extensive attention from scholars [8,9,10]. CIR reflects the fluctuation and fading of the signal in the channel environment, so the channel parameters of CIR are combined with joint likelihood function [11], machine learning [12], threshold comparison [13], and deep learning [14,15,16] to identify NLOS/LOS.

In this paper, a novel method for NLOS/LOS identification based on CIR combined with deep learning is proposed. This method aims to improve NLOS/LOS identification accuracy and reduce computational complexity. Our main contributions are summarized as follows:

1. A multilayer Convolutional Neural Network (CNN) combined with a Channel Attention Module (CAM) for the NLOS/LOS identification method is proposed. The method takes the One-dimensional CIR signal as input, uses three groups of convolution modules (Convolution + BN + ReLU + Max-pooling) and CAM for self-extraction of key features, and the global average pooling layer is used to replace the fully connected layer for feature integration and classification output, which achieves NLOS/LOS identification.
2. Two schemes are proposed on how to determine the specific structure of the CNN-CAM network and how to determine the optimal parameters. In the first scheme, the proposed CNN-CAM model is compared with CNN and CNN-CAM models with different structures, and it aims to select the optimal model structure for NLOS/LOS identification. In the second scheme, the effect of different learning rates and batches on the identification accuracy is compared experimentally for the proposed model, and it aims to determine the optimal parameters of the model.
3. A scheme on how to verify the superiority of the proposed CNN-CAM method is offered. Firstly, the public dataset of the European Horizon 2020 program project eWINE is visualized and analyzed to illustrate the feasibility of using this dataset for experiments. Then, comparative experiments of several machine learning and deep learning identification methods are conducted using the dataset to validate the state-of-the-art of the proposed CNN-CAM method.

The rest of the paper is organized as follows. Section 2 introduces the related work of other scholars in the field of CIR-based NLOS/LOS identification. Section 3 analyzes the key problems of NLOS/LOS and the performance of CIR in the NLOS/LOS environment. They provide new ideas for the design of the proposed model. Section 4 describes the details of the proposed NLOS/LOS identification method based on CNN-CAM. Section 5 performs a series of visualizations on the CIR dataset and designs various experiments to evaluate the performance of the proposed method. Section 6 summarizes the article and discusses the advantages and limitations of this approach and future work.

## 2. Related Work

As mentioned in Section 1, NLOS/LOS identification methods are classified into three categories. Among them, CIR-based NLOS/LOS identification methods have received extensive attention from scholars. In this paper, we focus on this class of methods.

The channel parameters proposed in the existing literature mainly include kurtosis, skewness, maximum amplitude, peak time, rise time, total energy, mean excess delay, root-mean-square (RMS) delay spread, saturation, peak-to-average ratio, received signal power, energy steep rise amplitude, false crests number, first path error, first path distance error, etc. [9,10]. Ref. [11] proposed a method to identify NLOS and LOS using three-channel parameters, kurtosis, RMS delay spread, and mean excess delay, as statistical information and by building a likelihood function. Marano et al. extracted six channel parameters from CIR waveforms, including kurtosis, received signal power, maximum amplitude, rise time, mean excess delay, and RMS delay spread, then used Least Squares Support Vector Machine (LS-SVM) for NLOS and LOS identification [12]. However, the method does not consider the correlation between channel parameters. Li et al. proposed a new method that takes the sum of peak time and rise time as a new channel parameter and combines it with the number of undetected peaks [13]. The method has a high identification accuracy when the threshold is selected appropriately, but it is prone to identification errors when the peak time and rise time differ significantly from the expected or when the threshold is selected incorrectly. NLOS/LOS identification is essentially a binary classification problem. Additionally, the above methods need to manually extract the channel parameters of the CIR for NLOS/LOS identification, which can lead to a system whose reliability and robustness cannot be guaranteed.

In recent years, with the rapid development of deep neural networks, NLOS/LOS identification methods based on deep learning have received extensive attention from scholars. This method uses CIR data as system input and accomplishes feature extraction employing model self-learning. Jiang et al. used invertible transform for denoising the CIR dataset (European Horizon 2020 Programme project eWINE) and used CNN models to identify NLOS. The identification accuracy of the method is up to 81.68% [14]. Jiang et al. trained and tested the Convolutional Neural Network and Long Short-Term Memory (CNN-LSTM) model using the CIR dataset (European Horizon 2020 Programme project eWINE) as input. The method achieves an identification rate of up to 82.14% [15]. Li et al. proposed a method that takes the real and imaginary parts of the original CIR and its Fourier transform as inputs and utilizes a three-channel Convolutional Neural Network and Bidirectional Long Short-Term Memory (CNN-BiLSTM) for identification. The article used public datasets (European Horizon 2020 Programme project eWINE) to verify and found that the proposed method has an identification accuracy of 85.71% and outperforms both LSTM and CNN-LSTM [16]. Pei et al. proposed a Fully Convolution Network (FCN) joint self-attention mechanism for NLOS/LOS identification. The method is validated using public datasets (European Horizon 2020 Programme project eWINE), and the proposed method is found to have the highest accuracy of about 88.24% compared to CNN, LSTM, CNN-LSTM, FCN, and LSTM-FCN [17]. However, this method is affected by the quality and quantity of the training datasets. Refs. [18,19] proposed a method that converts one-dimensional CIR data into two-dimensional images and uses deep learning networks for identification. Its accuracy is affected by image size and inefficient operation.

To address the problems in the above methods, such as manual feature extraction leads to incomplete database content of candidate classification features, difficulty in selecting appropriate thresholds in multiple scenarios, and low recognition rates of other neural network methods. The NLOS/LOS identification method based on multilayer CNN combined with CAM is proposed. In other terms, embedding CAM in the CNN module reduces the redundant information generated in feature self-extraction and improves the characterization ability of the CNN. The input layer reduces the computational complexity by replacing the two-dimensional feature map with a one-dimensional feature map. The traditional CNN is improved by adding a batch normalization (BN) layer and a Rectified Linear Unit (ReLU) between the convolutional and pooling layers to speed up the convergence. Moreover, a Global Average Pooling (GAP) layer is chosen instead of a fully connected layer to reduce the training parameters and improve the model’s generalization ability. It obtains better identification results using CNN-CAM compared with other ways. The specific details of the proposed method are described in Section 4.

## 3. Preliminaries

In this section, the variability of UWB-ranging performance in the NLOS/LOS environment is tested. In addition, the performance of CIR is analyzed using the IEEE802.15.4a standard channel model.

### 3.1. NLOS/LOS Problem Statement

**Figure 1:**
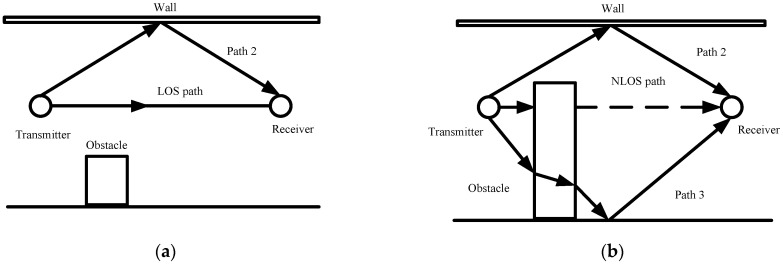
Schematic diagram of LOS and NLOS propagation: (**a**) LOS paths; (**b**) NLOS paths.

In complex indoor environments, the obstacles between the transmitter and receiver result in signal propagation through multiple paths. Among them, the LOS path means that the signal propagates directly between the transmitter and the receiver. The NLOS path means that the signal reaches the receiver by reflection, diffraction, and scattering. The LOS and NLOS propagation schematic is shown in Figure 1.

It can be seen from Figure 1a that the direct physical link between devices is not obscured in the LOS environment. The distance between the devices can be accurately estimated using the propagation time of the UWB direct path signal. It can be seen from Figure 1b that the direct physical link is obscured, causing the direct path signal to be curtailed and not accurately received. The UWB signals are affected by reflection, refraction, and scattering from obstacles during propagation, resulting in the additional distance. In this case, there is a delay in signal arrival time, which leads to reduced ranging accuracy.

**Figure 2:**
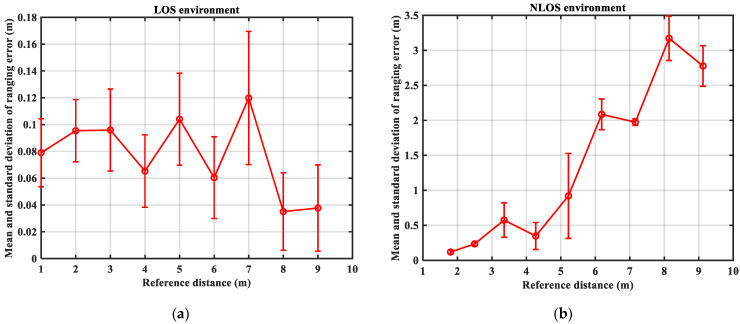
Graph of ranging error at different distances: (**a**) LOS environment; (**b**) NLOS environment.

UWB-based Indoor Positioning System (IPS) uses distance information from different channels to calculate positioning results. To test the variability of UWB ranging performance in LOS and NLOS environments, ranging experiments were conducted in LOS/NLOS environments. The ranging error results for the LOS and NLOS environments are provided in Table 1 and Table 2, respectively. The bar graph of ranging error at different distances in LOS/NLOS environments is shown in Figure 2. In addition, the mean and standard deviation of the ranging errors for each reference distance in both the LOS and NLOS environments are the result of calculations using 192 data.

Table 1 and Table 2 and Figure 2 show that the range error between the anchor node and the target node in the LOS environment is not large, and its mean and standard deviation of the range error do not exceed 0.1198 m and 0.0497 m, respectively. However, the mean value of the range error in the NLOS environment is not less than 0.3476 m, and the standard deviation of the range error is not less than 0.0318 m. Therefore, it is necessary to perform NLOS identification before positioning to achieve better positioning results.

### 3.2. CIR Performance Analysis

The CIR is the sum of the received pulses obtained by evaluating the correlation between the cumulative incoming samples and the expected lead sequence [20].

**Figure 3:**
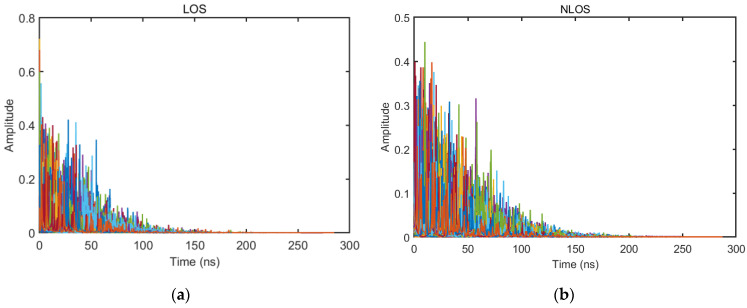
CIR waveform in LOS/NLOS environment: (**a**) LOS CIR waveform; (**b**) NLOS CIR waveform.

**Figure 4:**
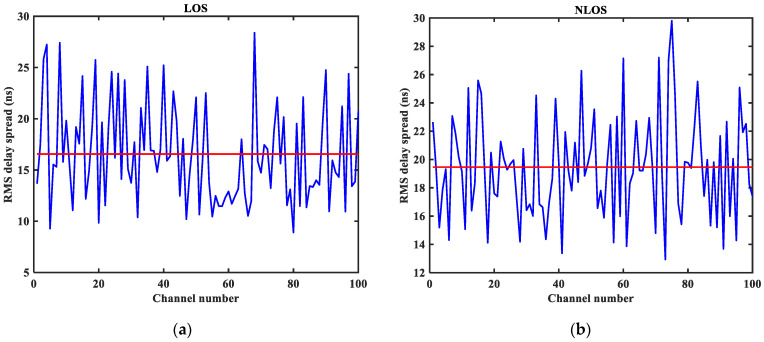
Root mean square (RMS) delay spread in LOS/NLOS environment: (**a**) LOS RMS delay spread; (**b**) NLOS RMS delay spread.

**Figure 5:**
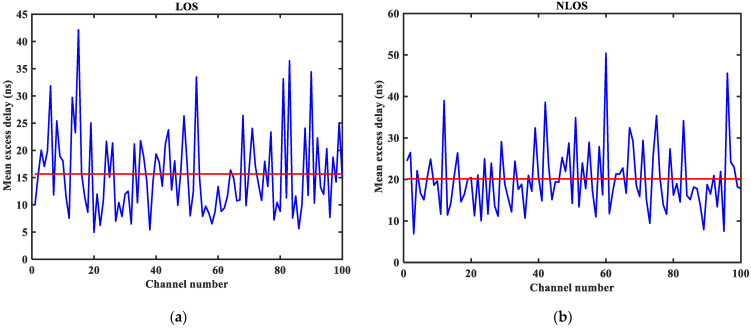
Mean excess delay in LOS/NLOS environment: (**a**) LOS mean excess delay; (**b**) NLOS mean excess delay.

**Figure 6:**
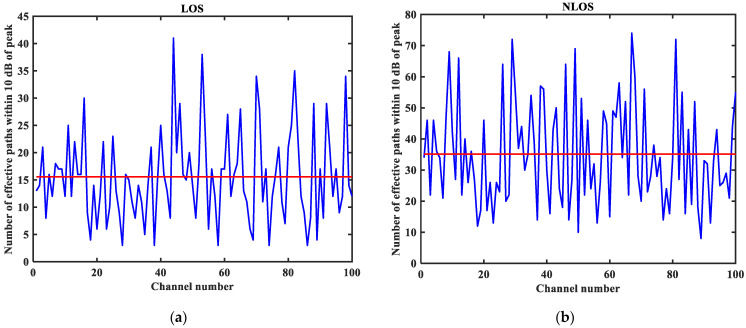
Number of effective paths with a peak within 10 dB in LOS/NLOS environment: (**a**) LOS number of effective paths with a peak within 10 dB; (**b**) NLOS number of effective paths with a peak within 10 dB.

**Figure 7:**
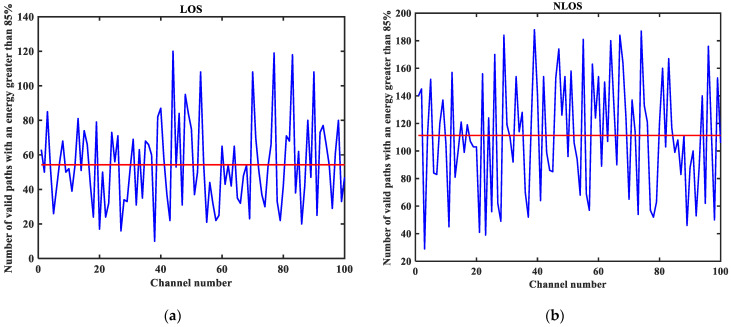
The number of valid paths with energy greater than 85% in the LOS/NLOS environment: (**a**) LOS number of valid paths with energy greater than 85%; (**b**) NLOS number of valid paths with energy greater than 85%.

**Figure 8:**
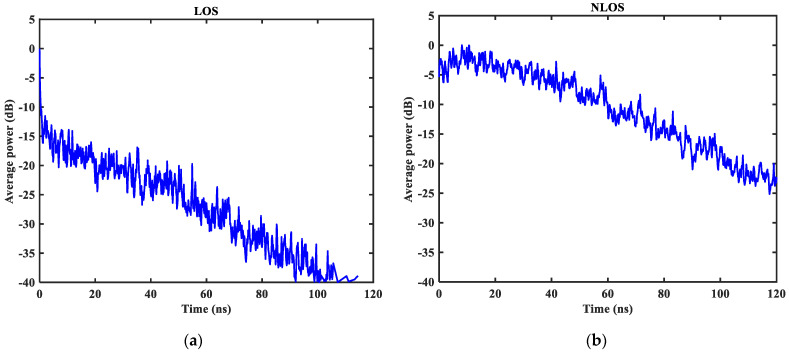
Average power decay curve in LOS/NLOS environment: (**a**) LOS average power; (**b**) NLOS average power.

As shown in Figure 3, Figure 4, Figure 5, Figure 6, Figure 7 and Figure 8 (the red lines in Figure 4, Figure 5, Figure 6 and Figure 7 represent their respective mean values), the IEEE802.15.4a standard channel model is chosen to analyze the CIR performance in LOS and NLOS environments. Firstly, the indoor residential LOS environment (CM1) and the indoor residential NLOS environment (CM2) are selected. Secondly, the corresponding specific parameters are selected according to the channel environment [21]. In addition, the continuous pulse function of the channel is realized according to the specific parameters, and then the continuous pulse function is discretized. Finally, RMS delay spread, mean excess delay, number of effective paths with a peak within 10 dB, number of valid paths with energy greater than 85%, and average power are calculated.

From Figure 3, it can be seen that the signal attenuation is relatively slow in the LOS environment, and the peak value of the CIR waveform is high, which is because the signal can reach the receiver through a direct path. However, in the NLOS environment, the signal amplitude is relatively tiny and decays quickly due to the obstacle blockage. In the LOS environment, the RMS delay spread, and the mean excess delay are shorter than the average time required in the NLOS environment. In the case of the same number of channels, the average values of the number of effective paths with peaks within 10 dB and the number of effective paths with energy greater than 85% are smaller in the LOS environment than in the NLOS environment. A comparison of the average power attenuation curves shows that the NLOS environment takes longer than the LOS environment when the receiver receives a signal with the same attenuation power.

In summary, the performance of CIR in NLOS and LOS environments is significantly different, so the deep learning method can directly use CIR as the input vector for NLOS /LOS identification.

## 4. Method

This paper aims to build a real-time NLOS/LOS identification method with a high recognition rate, high environmental applicability, and low computational complexity. The CNN combined with CAM for NLOS/LOS identification method is proposed. In this section, the related theories of CNN and CAM parts are introduced, and the proposed CNN-CAM network architecture and identification steps are described in detail.

### 4.1. CNN Theory

CNN is a feed-forward neural network inspired by natural biological visual cognitive mechanisms [22], which performs multiple convolution and pooling operations on the input data using multiple filters to obtain high-level features inside the data [23]. The structure of One-dimensional CNN mainly consists of convolution layers, pooling layers, and fully connected layers.

The convolution layer performs convolutional operations by sliding convolution kernels, and the output of these kernel filters is usually fed into the activation function to extract features. The one-dimensional CNN formula is as in Equation (1):

(1) $$y_{j}^{l}=f\left(\sum_{i=1}^{N}w_{ij}^{l}*x_{i}^{l-1}+b_{j}^{l}\right)$$

where ∗ indicates the convolution calculation; N is the number of kernel in (l−1)th layer; $x_{i}^{l-1}$ is the i feature mapping of the (l−1)th layer; $b_{j}^{l}$ is the bias of the jth convolution kernel of the lth layer; w is the weight; $y_{j}^{l}$ is the feature map representing the output of the jth convolution of the lth layer; f(·) represents the nonlinear activation function.

The activation function can enhance the nonlinear expression ability of the model. In this paper, we use the ReLU function, which enables neurons with sparse activation. The expression is shown in Equation (2).

(2) $$f_{ReLU}(x)=\max(0,x)$$

To further reduce the training parameters, the pooling or subsampling operation is often required, usually using Max-pooling or average pooling to compress the data in the sliding region. In this paper, Max-pooling is used, and the process is represented by Equation (3):

(3) $$p_{i}^{l+1}(j)=\underset{(j-1)\omega +1\leq t\leq j\omega }{\max}\{q_{i}^{l}(t)\}$$

where $q_{i}^{l}(t)$ is the value of the tth neuron corresponding to the ith feature; ω is the pooling layer width; $p_{i}^{l+1}(j)$ is the (l+1)th layer neuron value.

After alternating convolutional and pooling layers several times, the pooling layers are flattened and connected to one or more fully connected layers to achieve classification.

### 4.2. Attention Mechanism

**Figure 9:**
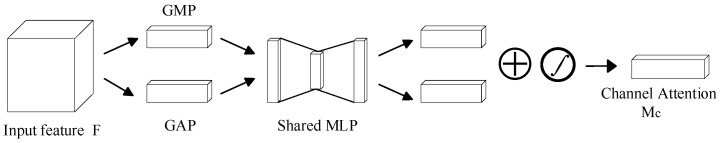
CAM structure diagram.

Attention mechanisms have been widely used in natural language processing, data prediction, hydroacoustic identification, image segmentation, etc. Compared to deep learning network architectures, the attention mechanism is a lightweight module that tunes the network parameters by generating and assigning weights and trains the network to focus on key information to improve accuracy [24,25]. In this paper, the CAM is added to the CNN, and its structure is shown in Figure 9. Firstly, the input feature maps are transformed Into two one-dimensional vectors by Global Max Pooling (GMP) and GAP, respectively. Then, they are passed through a shared Multilayer Perceptron (MLP). Finally, to obtain the weight values of the channels corresponding to the feature maps, the two output terms of the MLP are summed by channel and normalized using the activation function sigmoid.

The corresponding theoretical Equation is (4):

(4) $$\begin{array}{cc} M_{c}(F) & =\sigma (MLP(AvgPool(F))+MLP(MaxPool(F)))\\ & =\sigma \left(W_{1}\left(W_{0}\left(F_{\mathrm{avg}}^{c}\right)\right)+W_{1}\left(W_{0}\left(F_{\max}^{c}\right)\right)\right) \end{array}$$

where σ denotes the sigmoid function, both $W_{0}$ and $W_{1}$ are weights of MPL, and $F_{\mathrm{avg}}^{c}$ and $F_{max}^{c}$ are the characteristics of GAP and GMP, respectively [26].

### 4.3. NLOS/LOS Identification Method Based on CNN-CAM

In this section, the advantages of the fusion of both CNN and CAM are explained, the CNN-CAM network structure is constructed, the parameters of each layer and the identification steps are described in detail, and the performance evaluation metrics are given.

#### 4.3.1. CNN-CAM Network Architecture

**Figure 10:**
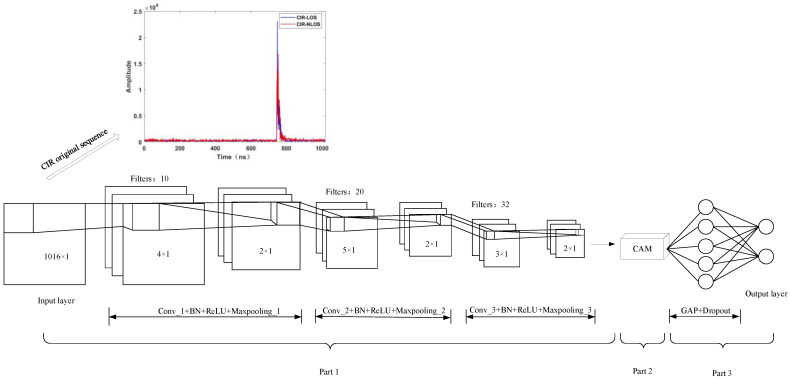
CNN-CAM network architecture.

The CIR of UWB can be considered a time series, and there is a correlation between the before and after data under LOS conditions. The data under NLOS conditions have apparent differences. Therefore, the CNN network is introduced into the paper, which has more advantages in learning the structural relationship between CIR data. However, traditional CNN takes the same way to convolve each channel of the feature map. In fact, different channels carry different importance of information, so processing each channel in the same way will degrade the accuracy of the network. CAM is a lightweight and universal module. It can not only assign different weights to input features to highlight important features and suppress useless feature responses but also integrates seamlessly with any CNN architecture for end-to-end training. Its advantages have been validated on different classification and detection datasets. Therefore, this paper embeds CAM in the multilayer CNN and builds the NLOS/LOS identification system based on CNN-CAM. The CNN-CAM network architecture is shown in Figure 10, and its specific parameters are shown in Table 3.

As can be seen from Figure 10 and Table 3, the CNN-CAM network architecture consists of three parts.

The first part contains three convolution layers, and each convolution module consists of a convolution layer, a BN layer, a ReLU function, and a Max-pooling layer. The input vector was the CIR data with the size 1016 × 1. Moreover, the first convolution layer uses the 4 × 1 convolution kernel with a number of 10 to perform the initial feature extraction operation. The second convolutional layer uses the 5 × 1 convolution kernel with a number of 20. The third convolutional layer uses the 3 × 1 convolution kernel with a number of 32 to mine deeper information from the output features of the upper layer. The stride size of all convolution layers is 2. The sizes of the convolution kernels used in this network’s first and second convolution layers were obtained from the literature [11,12]. In the third convolutional layer, we have used a 3 × 1 convolution kernel. This convolution kernel was obtained after conducting experiments on the effect of different sizes of convolutional kernels on the identification accuracy. In the experiment, 1 × 1, 2 × 1, 3 × 1, 4 × 1, and 5 × 1 are selected as the selection list of convolution kernel size. The BN can make the input samples become normally distributed with mean 0 and variance 1, thus solving the problem of slow learning speed due to the scattered distribution of sample features. Therefore, the paper adds BN layers after each convolutional layer to speed up the model training. And chooses ReLU as the activation function after BN layers. The pooling layer not only reduces the size of the parameter matrix but also allows filtering operations for additional noise introduced by the CIR signal under the influence of hardware circuits, transmission paths, NLOS receiving surfaces, and other factors. Therefore, the paper adds a 2 × 1 Max-pooling layer with a step size of 2 after each convolution layer.

The second part adds CAM on top of the above to further enhance the feature extraction capability of the model. The Max-pooling layer in the third convolution module is used as the input of CAM, and the GMP and GAP operations are performed, respectively. Subsequently, the two pooling layers sequentially perform a convolution of 1 × 1 of the number 8, the BN layer, the ReLU function, and the convolution of 1 × 1 of the number 32. Finally, the operations of superposition, sigmoid function, and multiplication are experienced.

The third part uses a convolution layer to transform the data dimensions and a GAP layer instead of a fully connected layer for feature integration. Finally, the softmax activation function accomplishes the identification of NLOS/LOS.

#### 4.3.2. NLOS/LOS Identification Process

**Figure 11:**
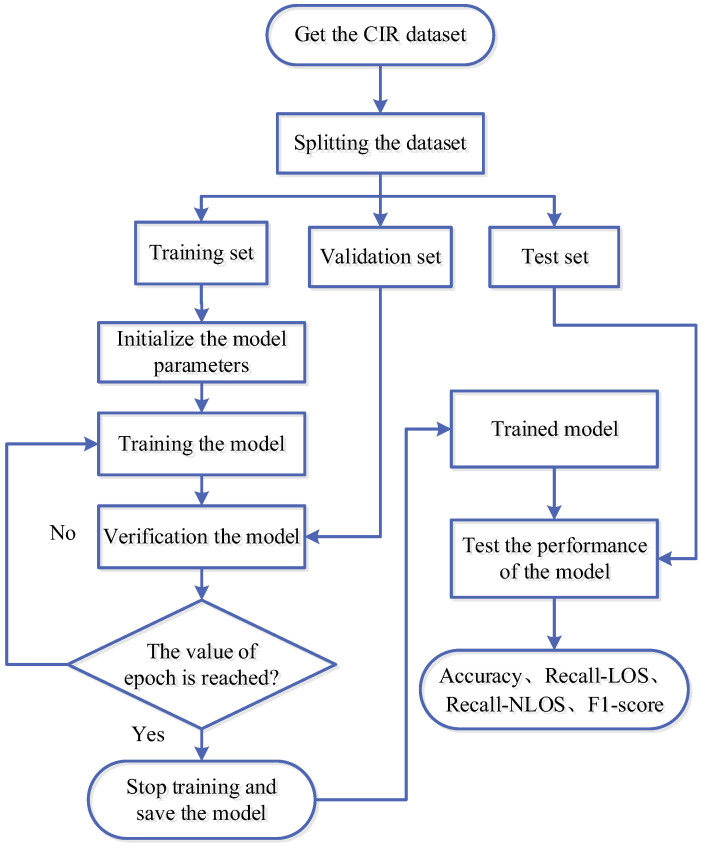
NLOS/LOS identification process of CNN-CAM.

The NLOS/LOS identification process based on CNN-CAM is shown in Figure 11. The steps are as follows:

1. The obtained CIR data is divided into training sets, validation sets, and test sets in the ratio of 7:2:1.
2. Train the CNN-CAM model with the training sets and validate the performance of the trained model with the validation sets. Furthermore, the trained model is saved when the epoch is reached.
3. The trained model is tested with test sets to obtain the final NLOS/LOS identification result.

In this paper, the order of the training set is randomly disrupted with the aim of improving the robustness of the model. In addition, the Adam optimizer is used for each training period, the learning rate decay period is set to 10, and the learning rate decay is 0.5 times the original.

In addition, to evaluate the performance of the proposed model, we use four metrics: Accuracy, LOS recall, NLOS recall, and F1-score. As shown in Equations (5), (6), (7), and (8), respectively [8,9].

(5) $$Accuracy=\frac{TP+TN}{TP+TN+FP+FN}$$

(6) $$Recall\text{-}LOS=\frac{TP}{TP+FN}$$

(7) $$Recall\text{-}NLOS=\frac{TN}{TN+FP}$$

(8) $$F1\text{-}score=\frac{2TP}{2TP+FN+FP}$$

where TP is the number of data correctly identified as LOS, TN is the number of data correctly identified as NLOS, FN is the misjudgment data for LOS, and FP is the misjudgment data for NLOS.

## 5. Results and Discussion

In this section, firstly, the dataset was briefly described and visualized. Secondly, experiments on model parameters are designed to determine the optimal values of learning rate and batch size. Finally, to verify the advancedness and effectiveness of the model, experiments with different structural models and different identification methods were conducted.

### 5.1. Visual Analysis of Datasets

**Figure 12:**
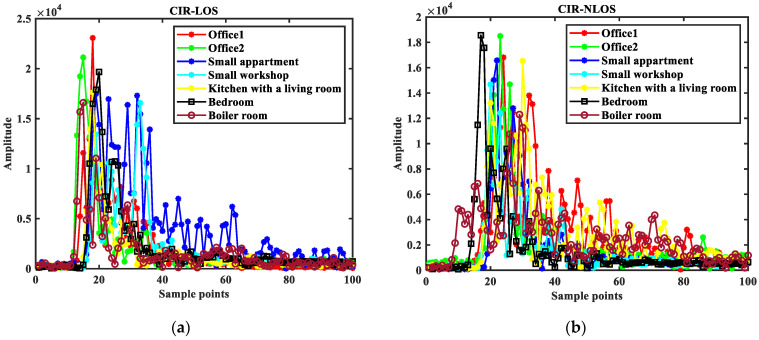
Schematic diagram of CIR sampling points in the seven environments (731 ns–830 ns): (**a**) LOS environment; (**b**) NLOS environment.

**Figure 13:**
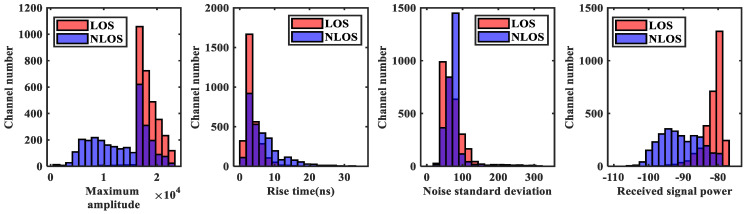

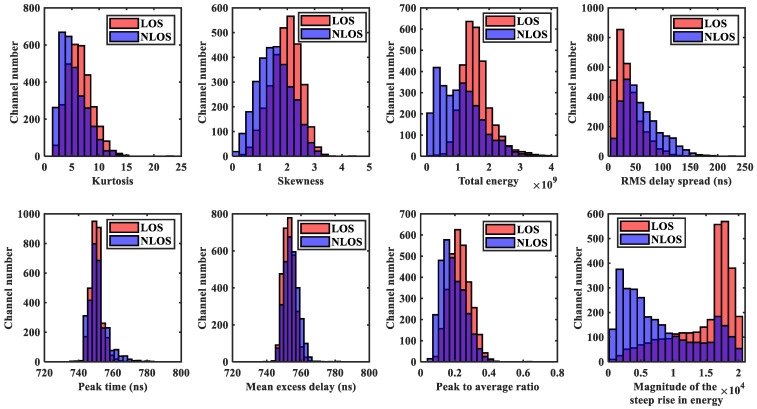
Numerical distribution of signal characteristic parameters in LOS and NLOS environments (Purple color represents the portion where the number of channels are overlapped).

The public dataset was used for experiments [27]. The data are measured in seven indoor scenarios, including office 1, office 2, a small apartment, a small workshop, a kitchen with a living room, a bedroom, and a boiler room. In addition, 3000 LOS and 3000 NLOS channel measurements were collected in each scenario. Figure 12 shows the CIR sampling points in the seven environments, and Figure 13 shows the numerical distribution of the signal characteristic parameters in LOS and NLOS environments.

From Figure 12, it can be seen that the CIR waveform is not clearly distinguished in each environment, reflecting the complexity of this dataset. From Figure 13, it can be seen that the maximum amplitude, rise time, noise standard deviation, received signal power, kurtosis, skewness, total energy, RMS delay spread, peak time, mean excess delay, peak-to-average ratio, the amplitude of the steep rise in energy have relatively high overlap. And it shows that the difference between LOS and NLOS datasets is not apparent.

In summary, the dataset selected in this paper has the test conditions to verify the proposed method.

### 5.2. Experiments and Results

In NLOS/LOS identification based on CNN-CAM, there are mainly three parts of the experiment. Firstly, we conducted experiments for different sizes of learning rates and training batches to select the appropriate parameters. In addition, we designed six models with different structures to verify the effectiveness of adding the CAM and the three-layer convolution module (Convolution + BN + ReLU + Max-pooling). Finally, to verify the model’s advancedness, we compare the proposed method with the state-of-the-art NLOS/LOS identification methods, such as the identification method of joint machine learning of channel parameters and the identification method of other deep learning.

#### 5.2.1. Parameter Analysis

**Figure 14:**
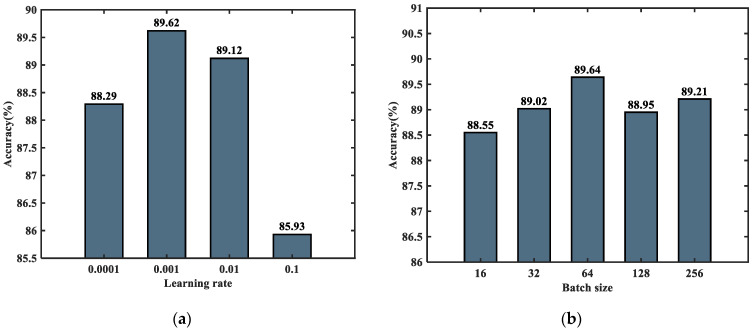
Graph of the effect of parameters on model performance: (**a**) Model performance with different learning rates; (**b**) Model performance with different batch sizes.

Model parameters play an essential role in the performance of the network. Suitable parameters not only improve the convergence speed in the training phase but also help to achieve better classification results. Different learning rates and batch sizes were selected to find the best value of learning rate and batch sizes for performance comparison, as shown in Figure 14a,b.

The learning rate refers to the magnitude of each update of the parameters. If the learning rate is too low, the convergence speed of the network will be slow. If the learning rate is too large, the optimized parameters will fluctuate repeatedly near the optimal value, making network convergence difficult. From Figure 14a, it can be seen that there is a relationship between the model’s accuracy and the learning rate, with a maximum value of 89.62% achieved at 0.001. However, the accuracy rate decreases when the learning rate decreases to 0.0001. Therefore, the best learning rate determined using the CNN-CAM model is 0.001.

When the training batch is too small, the difference in the samples leads to an extensive range of statistical characteristics of the batch, which makes the direction of gradient descent unstable, so the identification accuracy fluctuates wildly. When the training batch is too large, due to the limitation of the training set size, its number of gradient descents is too tiny to find the global optimal solution easily. At this time, the statistical characteristics of large-scale samples are considered, which do not accurately represent the direction of gradient update in the training set. From Figure 14b, it can be observed that the model achieves the highest accuracy when the training batch size is set to 64, suggesting that optimal results can be achieved by setting the model batch size to 64.

#### 5.2.2. Performance Analysis

Various comparative experiments were designed to compare the proposed model’s performance: different structural models, identification methods for joint machine learning of feature parameters, and other deep learning identification methods. In addition, we use four metrics: Accuracy, Recall-LOS, Recall-NLOS, and F1-score to evaluate the model performance.

(a) Comparative Experiments of Different Structural Models

**Figure 15:**
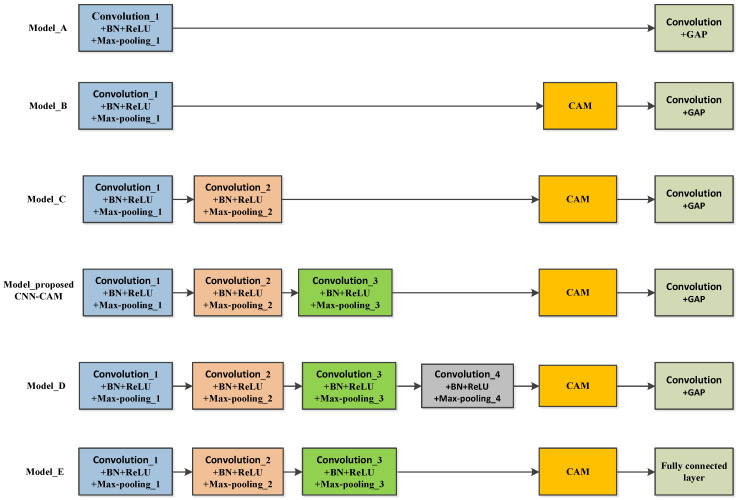
Models of different structures.

**Figure 16:**
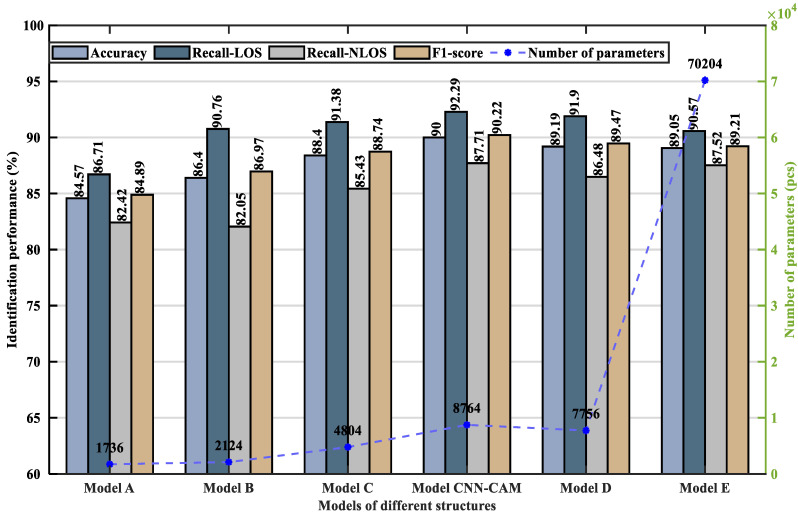
Model performance comparison chart for different structures.

To verify the validity of the proposed model, the paper constructed six models with different structures and analyzed their performance, as shown in Figure 15 and Figure 16 and Table 4, respectively.

As can be seen from Figure 15 and Figure 16 and Table 4, Model A did not add CAM, and its accuracy was the lowest. Model B consists of a layer of convolutional modules (Convolution + BN + ReLU + Max-pooling), CAM, and “Convolution + GAP”. The accuracy leveled off when the training model increased with the number of iterations. At this point, the accuracy of model B has increased by 1.83%, the LOS recall has increased by 4.05%, the NLOS recall has remained essentially unchanged, and the F1-score has increased by 2.08%, indicating that the addition of CAM can improve the identification accuracy. Model C adds a layer of convolution module (Convolution + BN + ReLU + Max-pooling) on the basis of Model B. Its accuracy is 88.40%, LOS recall is 91.38%, NLOS recall is 85.43%, F1-score is 88.74%, and the number of parameters is 4804. The proposed CNN-CAM model is based on model B with the addition of a two-layer convolution module (Convolution + BN + ReLU + Max-pooling), which achieves an accuracy of 90.00%, a LOS recall of 92.29%, an NLOS recall of 87.71%, an F1-score of 90.22%, and a parameter count of 8764. Model C and the proposed CNN-CAM model increase the number of parameters, but improve the accuracy by 2.00% and 3.60%, the LOS recall by 0.62% and 1.53%, the NLOS recall by 3.38% and 5.66%, and the F1-score by 1.77% and 3.25%, respectively, when compared to Model B. It indicates that adding the convolution module can improve the identification accuracy. Model D continues to add a layer of convolution module, which reduces the recognition rate, verifying the feasibility of using a three-layer convolution module. Model E replaces the GAP in the proposed model with a fully connected layer. Every neuron in the fully connected layer is linked to all the neurons in the previous layer, and the number of trainable parameters increases from 8764 to 70,204, which causes an overfitting phenomenon.

In summary, by analyzing the performance of the above models with different structures, it is found that the CNN-CAM model proposed in this paper ensures good fitting performance with fewer trainable parameters and the highest model accuracy, which verified the effectiveness of the CNN-CAM model proposed.

(b) Comparison Experiments of Different Identification Methods

To verify the advancedness of the proposed CNN-CAM model, CNN-LSTM [15], CNN-SVM, and Random Forest (RF) models are selected for comparison with the model proposed in this paper. The CNN-SVM model is based on the feature vectors extracted from the CNN fully connected layer as input, and the kernel function of the SVM classifier is the radial basis function (RBF). The RF (single feature) approach takes a kurtosis feature as input and uses RF for identification. RF (multiple features) method is used to extract 12 feature parameters of CIR data, including kurtosis, skewness, total energy, RMS delay spread, peak time, mean excess delay, peak-to-average ratio, amplitude of the steep rise in energy, maximum amplitude, rise time, noise standard deviation, and received signal power, and then use RF to identify them. In addition, the paper sets the number of decision trees in the RF algorithm to 100, and the minimum number of samples of leaf nodes is set to 1. A comparison of the performance of different identification methods is shown in Table 5.

As shown in Table 5, the CNN-CAM model proposed in this paper achieves 90.00% accuracy, 92.29% LOS recall, 87.71% NLOS recall, and 90.22% F1-score. In terms of accuracy, compared to CNN-LSTM, CNN-SVM, RF (single feature), and RF (multiple features), the accuracy of the CNN-CAM model is improved by 5.06%, 3.88%, 35.48%, and 2.57%, respectively. In terms of LOS recall, CNN-CAM has a significant improvement effect compared to CNN-LSTM, CNN-SVM, RF (single feature), and RF (multiple features), with a minimum improvement of 6.62% and a maximum of 38.21%. In terms of NLOS recall, CNN-CAM outperforms CNN-LSTM, CNN-SVM, and RF (single feature) and is slightly lower than RF (multiple features). However, the use of multiple feature parameters for identification improves the NLOS recall, but the accuracy and stability are affected because there is some irrelevant and redundant information between the artificially extracted features. In terms of F1-score, CNN-CAM shows significant improvement compared to CNN-LSTM, CNN-SVM, RF (single feature) and RF (multiple features). Furthermore, the lowest improvement is 2.37%, and the highest is 36.12%. In summary, the CNN-CAM network model proposed in this paper extracts features with higher sensitivity and has obvious performance improvement effects compared to the neural network models and machine learning models in existing studies. Moreover, it is more suitable for NLOS/LOS identification.

## 6. Conclusions

In this paper, firstly, the features of CIR data in LOS and NLOS environments are analyzed in detail, and the factors affecting range are identified. Then, aiming at the problem of low accuracy and poor environmental adaptability of existing UWB CIR identification methods, the NLOS/LOS identification method based on multilayer CNN combined with CAM is proposed. The method takes the one-dimensional CIR signal as input, uses three groups of convolution modules (Convolution + BN + ReLU + Max-pooling) and CAM for feature self-extraction, and uses a GAP for feature integration to achieve NLOS/LOS identification. A Max-pooling layer is used to achieve the filtering operation for the extra noise introduced by the CIR signal. The overfitting phenomenon is avoided by replacing the fully connected layer with the GAP layer, which reduces the parameters by 87.52% compared to Model E. In addition, to compare the proposed model’s performance, a variety of comparative experiments were designed using public datasets: different structural models, identification methods for joint machine learning of feature parameters, and other deep learning identification methods. It is found that the proposed model has 90.00% accuracy, 92.29% LOS recall, 87.71% NLOS recall, and 90.22% F1-score, which achieves high identification results in NLOS/LOS identification of UWB and verifies the effectiveness and advancement of the model.

However, the inability to collect large datasets in real emergency scenarios limits the application scenarios of this network to some extent. Therefore, NLOS/LOS identification in the small sample case will be considered in future work.

## Figures and Tables

**Table 1:** LOS environment ranging error.

Reference Distance (m)	Mean of Ranging Error (m)	Standard Deviation of Ranging Error (m)
1.0000	0.0790	0.0253
2.0000	0.0954	0.0232
3.0000	0.0959	0.0306
4.0000	0.0653	0.0270
5.0000	0.1040	0.0343
6.0000	0.0604	0.0305
7.0000	0.1198	0.0497
8.0000	0.0351	0.0289
9.0000	0.0377	0.0322

**Table 2:** NLOS environment ranging error.

Reference Distance (m)	Mean of Ranging Error (m)	Standard Deviation of Ranging Error (m)
1.8023	0.1186	0.0275
2.5000	0.2348	0.0318
3.3541	0.5747	0.2456
4.272	0.3476	0.1922
5.2202	0.9203	0.6075
6.1847	2.0852	0.2191
7.159	1.9741	0.0469
8.1394	3.1719	0.3178
9.1241	2.7760	0.2898

**Table 3:** CNN-CAM specific parameters table.

Network Composition	Designation	Parameter
Part 1	Sequence Input	1016 × 1 × 1
	Convolution_1 (stride)	4 × 1 × 10 (2)
	BN, ReLU	——
	Max-pooling_1 (stride)	2 × 1 (2)
	Convolution_2 (stride)	5 × 1 × 20 (2)
	BN, ReLU	——
	Max-pooling_2 (stride)	2 × 1 (2)
	Convolution_3 (stride)	3 × 1 × 32 (2)
	BN, ReLU	——
	Max-pooling_3 (stride)	2 × 1 (2)
Part 2	GMP, GAP	——
	Convolution_4/Convolution_6	1 × 1 × 8
	BN, ReLU/BN, ReLU	——
	Convolution_5/Convolution_7	1 × 1 × 32
Part 3	Convolution	1 × 1 × 128
	GAP	——
	Dropout	0.5
Training	Epoch	25
	Learning rate	0.001
	Batch size	64

**Table 4:** Comparison table of model performance of different structures.

Model Names	Number of Parameter	Accuracy (%)	Recall-LOS (%)	Recall-NLOS (%)	F1-Score (%)
Model_A	1736	84.57	86.71	82.42	84.89
Model_B	2124	86.40	90.76	82.05	86.97
Model_C	4804	88.40	91.38	85.43	88.74
Model_proposed CNN-CAM	8764	90.00	92.29	87.71	90.22
Model_D	7756	89.19	91.90	86.48	89.47
Model_E	70,204	89.05	90.57	87.52	89.21

**Table 5:** Performance comparison of different identification methods.

Methods	Accuracy (%)	Recall-LOS (%)	Recall-NLOS (%)	F1-Score (%)
CNN-LSTM	84.94	84.91	84.97	84.93
CNN-SVM	86.12	85.67	86.57	86.06
RF (single feature)	54.52	54.08	54.96	54.10
RF (multiple features)	87.43	85.52	89.59	87.85
CNN-CAM proposed	90.00	92.29	87.71	90.22

## Data Availability

Data is not provided temporarily due to restrictions, such as privacy or ethics.
